# *Fatty acid desaturase* insertion-deletion polymorphism rs66698963 predicts colorectal polyp prevention by the *n–*3 fatty acid eicosapentaenoic acid: a secondary analysis of the seAFOod polyp prevention trial

**DOI:** 10.1016/j.ajcnut.2024.06.004

**Published:** 2024-06-13

**Authors:** Ge Sun, Yan Ning Li, John R Davies, Robert C Block, Kumar SD Kothapalli, J Thomas Brenna, Mark A Hull

**Affiliations:** 1 Leeds Institute of Medical Research, University of Leeds, Leeds, United Kingdom; 2 Department of Pediatrics, Dell Pediatric Research Institute, Dell Medical School, The University of Texas at Austin, TX, United States; 3 Department of Nutritional Sciences, The University of Texas at Austin, Austin, TX, United States; 4 Department of Public Health Sciences, University of Rochester, Rochester, NY, United States; 5 Cardiovascular Division of the Department of Medicine, University of Rochester, Rochester, NY, United States; 6 Center for Community Health and Prevention, University of Rochester, Rochester, NY, United States

**Keywords:** arachidonic acid, eicosapentaenoic acid, *fatty acid desaturase*, genetic polymorphism, highly unsaturated fatty acid, polyp

## Abstract

**Background:**

A *fatty acid desaturase* (*FADS*) insertion-deletion (Indel) polymorphism (rs66698963) influences the expression of *FADS1*, which controls the synthesis of *n–*6 highly unsaturated fatty acid (HUFA) arachidonic acid (AA). The anti-inflammatory activity of the *n–*3 HUFA eicosapentaenoic acid (EPA) may be explained by competition with AA for proinflammatory lipid mediator synthesis. A precision medicine approach based on stratification by *FADS* Indel genotype could identify individuals, who benefit from greatest disease risk reduction by *n–*3 HUFAs.

**Objectives:**

We tested the hypothesis that the *FADS* insertion (I) allele predicts colorectal polyp risk reduction in a secondary analysis of the randomized, placebo-controlled, 2×2 factorial seAFOod polyp prevention trial of EPA 2000 mg daily and aspirin 300 mg daily for 12 mo (ISRCTN05926847).

**Methods:**

Participant Indel genotype was determined by polymerase chain reaction (PCR) blind to trial outcomes. Colorectal polyp outcomes were included in negative binomial (polyp number) and logistic (polyp detection rate [PDR; percentage with one or more polyps]) regression models comparing each active intervention with its placebo. Presence of ≥1 Indel I allele and an interaction term (I allele × active intervention) were covariates.

**Results:**

In 528 participants with colonoscopy and *FADS* Indel data, EPA use irrespective of Indel genotype, was not associated with reduced colorectal polyp number (incidence rate ratio [IRR]: 0.92; 95% confidence interval: 0.74, 1.16), mirroring original seAFOod trial analysis. However, the presence of ≥1 I allele identified EPA users with a significant reduction in colorectal polyp number (IRR: 0.50 [0.28, 0.90]), unlike aspirin, for which there was no interaction. Similar findings were obtained for the PDR.

**Conclusions:**

The *FADS* Indel I allele identified individuals, who displayed colorectal polyp prevention by EPA with a similar effect size to aspirin. Assessment of rs66698963 as a biomarker of therapeutic response to *n–*3 HUFAs in other populations and healthcare settings is warranted.

The seAFOod polyp prevention trial and STOP-ADENOMA study were registered at International Standard Randomised Controlled Trial Number registry as ISRCTN05926847.

## Introduction

Omega (*n*)–3 highly unsaturated fatty acids (HUFAs) have been investigated extensively for the prevention of solid cancers, including colorectal cancer (CRC) with mixed results [[1], [2], [3]]. A randomized trial of the *n–*3 HUFA eicosapentaenoic acid (EPA, C20:5*n*–3) in patients with familial adenomatous polyposis demonstrated that treatment with pure EPA-free fatty acid (FFA) 2000 mg daily for 6 mo reduced the number and size of rectal adenomatous polyps, which are established biomarkers of subsequent CRC risk [4], by ∼20% compared with placebo [5]. However, the efficacy of the same dose and formulation of EPA was modest and did not reach statistical significance (incidence rate ratio [IRR]: 0.91; 95% confidence interval [CI]: 0.79, 1.05) for the reduction in colorectal polyp number in the seAFOod 2 × 2 factorial polyp prevention trial of EPA and aspirin 300 mg daily in patients undergoing colonoscopy surveillance after removal of multiple sporadic colorectal polyps [6].

The mechanism of the anti-neoplastic activity of EPA is uncertain, but the strongest line of evidence suggests that EPA antagonizes protumorigenic signaling by competing with its *n–*6 HUFA homolog C20:4*n*–6 arachidonic acid (AA) as a substrate for cyclooxygenase (COX)-dependent synthesis of prostaglandin (PG) E_2_ [[7], [8]]. PGE_2_ and other eicosanoids derived from AA are proinflammatory and drive tumorigenesis, while the immediate precursor of AA, C20:3*n–*6 di-homo-gamma-linolenic acid (DGLA) is converted to the largely anti-inflammatory prostanoid PGE_1_. The rate-limiting step for endogenous AA synthesis from the dominant *n–*6 PUFA in western diets C18:2*n–*6 linoleic acid (LA) is Δ5-desaturation of DGLA by fatty acid desaturase 1 (FADS1) [[9], [10]]. FADS1 and FADS2 also contribute to *n–*3 HUFA synthesis from C18:3*n–*3 alpha-linolenic acid (ALA) [9].

We have previously characterized a functional 22 bp insertion-deletion (Indel) polymorphism (rs66698963) in intron 1 of the *FADS2* gene, which is 137 bp downstream of a sterol-response element (SRE) that controls *FADS1* expression [11]. *FADS1* and *FADS2* are oriented head-to-head and their expression may be coordinated. The regulatory Indel polymorphism is located in *FADS2* but primarily affects *FADS1* expression and function [11]; therefore, we refer to it as the “*FADS* Indel.” The *FADS* Indel is evolutionarily selective, probably based on ancestral dietary *n*–3 and *n–6* HUFA content, and controls circulating AA levels via *FADS1* in free-living humans across several diverse populations [[11], [12], [13]]. Homozygosity for the insertion (I) allele (a 22 bp tandem repeat) has the effect of increasing circulating AA levels by as much as 50% compared with deletion (D) allele (a single 22 bp copy) homozygotes [[12], [13]]. Therefore, the *FADS* Indel influences the balance between precursor DGLA and product AA, thereby controlling inflammatory balance.

Approaches to precision nutrition are being driven by the increased availability of personalized biomedical data through technological advances and declining measurement costs [14]. The host genome is one of the most prominent factors contributing to individual responses to nutrition and nutritional therapies, also including developmental origins of health and disease, lifestyle, and habitual dietary patterns [15]. A useful interpretation of the concept of precision nutrition is to aid the stratification of subgroups of the population in order to better define dietary requirements and recommendations [14]. This view comports with the precision medicine use of a response biomarker in a conventional randomized controlled trial setting to identify responders and nonresponders to a particular drug or nutrient intervention [16].

Based on the current mechanistic understanding that the *n–*3 HUFA EPA competes with its *n–*6 HUFA counterpart AA and inhibits protumorigenic signaling by downstream lipid mediators such as PGE_2_, we performed a secondary analysis of the seAFOod trial to test the hypothesis that the *FADS* Indel I allele, which drives FADS1-dependent AA synthesis and bioavailability, predicts colorectal polyp prevention efficacy of EPA.

## Methods

### Ethical approval and trial registration

This secondary analysis of the seAFOod polyp prevention trial was a component of the STOP-ADENOMA study, ethical approval for which was obtained from the London and Surrey Borders Research Ethics Committee (19/LO/1655). All seAFOod trial participants provided written, informed consent at registration for the use of trial data and biobank samples, including genetic studies, in approved projects after the end of the trial. The seAFOod trial and STOP-ADENOMA project were prospectively registered as ISRCTN05926847.

### seAFOod polyp prevention trial design

The seAFOod polyp prevention trial was a randomized, double-blind, placebo-controlled, 2 × 2 factorial trial of the CRC chemoprevention efficacy of EPA FFA 2000 mg daily and aspirin 300 mg daily in patients undergoing colonoscopy surveillance in the English Bowel Cancer Screening Program (BCSP) [6,17]. The intervention period between the index BCSP screening colonoscopy, at which all polyps were removed, and the first surveillance colonoscopy was 12 mo in individuals deemed high risk (defined as ≥5 polyps or ≥3 polyps, if one or more polyps were ≥10 mm in size). This duration has been associated with a similar degree of colorectal polyp risk reduction compared with a 3-year intervention period in previous aspirin polyp prevention trials [18]. seAFOod trial recruitment was inclusive to all individuals who were stratified as having a high risk for future colonoscopy surveillance by the English BCSP. However, the trial population was predominantly White European and male, with high levels (>80%) of excess body weight, mirroring the demographic of individuals undergoing English BCSP screening colonoscopy [6,17].

The primary outcome of the seAFOod polyp prevention trial was the adenoma (now more accurately termed polyp) detection rate (PDR; the percentage of individuals with one or more polyps [including both adenomatous and serrated polyps]) at surveillance colonoscopy 12 mo after clearance screening colonoscopy [6,17]. We and others now recognize that the secondary endpoint of colorectal polyp number is a better readout of polyp prevention efficacy in high-risk populations with multiple polyps [19,20], and have since used polyp multiplicity, in parallel with the PDR, in secondary seAFOod trial outcomes analyses [19,21,22]. Colorectal polyp number is the primary endpoint in the ongoing COLO-PREVENT polyp prevention trial of aspirin compared with aspirin and metformin [23].

### Laboratory methods

Buffy coat DNA samples from seAFOod participants were obtained as described [17,21]. Genotyping for the *FADS* Indel rs66698963 (I or D alleles) was performed by PCR and agarose gel electrophoresis on anonymized DNA samples, blinded to linked trial data [11,13].

Baseline and on-treatment (6 mo) red blood cell (RBC) membrane HUFA levels (as the percentage of total fatty acids) and plasma oxylipin (15-hydroxyeicosatetraenoic acid [HETE]) levels were measured by liquid chromatography-tandem mass spectrometry (LC-MS/MS), as described and have been published [6,22]. Plasma 15-HETE values were logarithmically transformed to achieve a normal distribution.

AA and LA, but not DGLA, were measured in the nine-fatty acid LC-MS/MS panel available for seAFOod RBC samples [17,24]. Therefore, LA, the dietary precursor of DGLA, was used for the product (AA)-precursor (LA) ratio used as the readout of FADS1 activity [13]. For investigation of the relationship between the *FADS* Indel genotype and the AA/LA ratio, as well as the plasma total (sum of *R*- and *S*- enantiomer concentrations [22]) 15-HETE concentration, data for participants who were either heterozygous (I/D) or homozygous (I/I) I carriers were combined due to the small number of I homozygotes in the predominantly White British seAFOod trial population [6]. Statistical significance of the difference between *FADS* Indel I allele carriers and D/D homozygotes was tested using either the Wilcoxon rank-sum test or t-test, based on the distribution of the data.

### Statistical analysis

The prespecified analysis of colorectal polyp number in the original seAFOod trial report was performed by Poisson regression, incorporating repeat colonoscopy after the baseline screening colonoscopy in the trial (as a potential confounder of polyp outcomes at the 12-month trial exit colonoscopy) and hospital research site, at which the colonoscopy was undertaken, as covariates [6]. We repeated this analysis for the smaller trial population that had an available *FADS* Indel genotype, to confirm that the effect sizes for a reduction in colorectal polyp number for both EPA and aspirin were similar to the original randomized trial population [6]. However, the distribution of individual posttreatment total colorectal polyp counts better fitted a negative binomial model [17], which has since been used in subsequent analyses of in-trial and post-trial colorectal polyp outcomes [19,21]. Therefore, negative binomial regression was used to explore the interaction between seAFOod trial treatment and *FADS* Indel genotype regarding colorectal polyp number. A logistic regression model was used to examine factors predicting the PDR.

All analyses were performed at the margins of the 2 × 2 factorial trial, ie, each active intervention (EPA or aspirin) was compared with its respective placebo, regardless of the other intervention [6]. Data are presented as the total colorectal polyp number and total PDR, as the sum of adenomatous and serrated (but not diminutive rectal hyperplastic) polyps, in keeping with the primary analysis of the seAFOod trial [6].

Consistent with the fatty acid and oxylipin analysis according to *FADS* Indel genotype, each regression model included the presence of ≥1 I allele (*FADS* Indel I carrier) as a binary covariate, as well as an interaction term for I carrier × active (either EPA or aspirin) intervention. Consistent with previous primary and secondary seAFOod polyp prevention trial analyses, we included repeat baseline colonoscopy (which some participants underwent for clinical indications such as polypectomy-site check or inadequate bowel preparation) as a covariate and adjusted for the hospital site where the research colonoscopy was performed [6,17,19]. Male sex was a covariate given the consistent relation with increased colorectal polyp risk in multiple previous studies [25,26]. Combined models were also derived that included EPA and aspirin as separate treatment terms given that post hoc trial analysis suggested that combination treatment was associated with reduced colorectal polyp recurrence compared with single agent use [19].

Data are reported as either the IRR and 95% CI for negative binomial models or the odds ratio (OR) and 95% CI for logistic regression models. All analyses were conducted using R Studio, version 2021.09.0 (Posit, Boston, MA).

## Results

**FIGURE 1:**
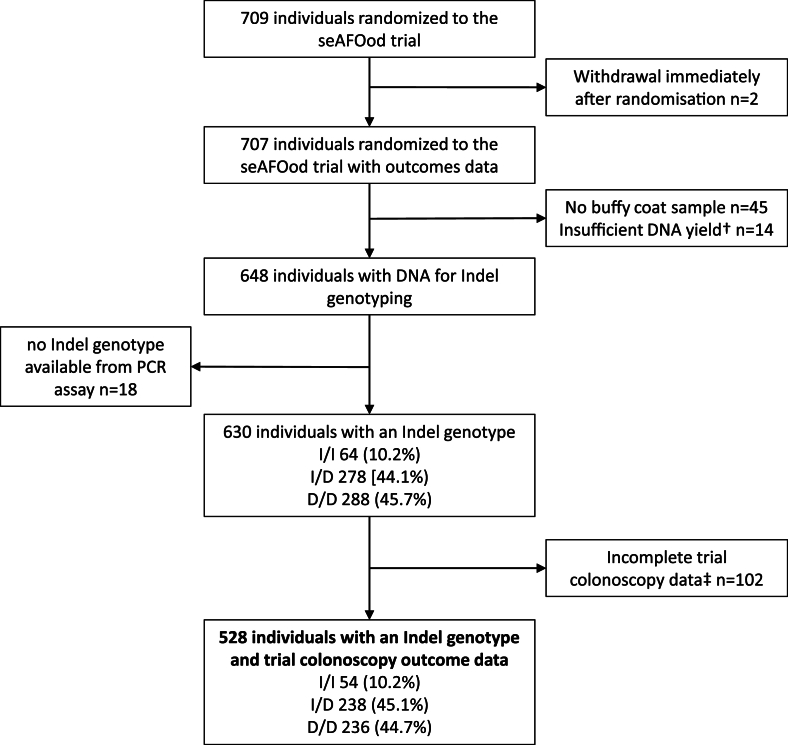
seAFOod trial participants and samples contributing to the treatment × *fatty acid desaturase* insertion-deletion genotype analysis. † indicates DNA <20 ng/mL. ‡ indicates incomplete trial colonoscopy data including 57 participants with no trial exit colonoscopy and 45 participants with missing data for repeat colonoscopy at baseline.

A *FADS* Indel genotype was available for 630 seAFOod trial participants (Figure 1). There were 64 (10.2%) I/I homozygotes in the predominantly White European seAFOod trial cohort [6,17]. Given the small number of I/I homozygotes, we combined I/I homozygotes and I/D heterozygotes for comparison with D/D individuals.

**FIGURE 2:**
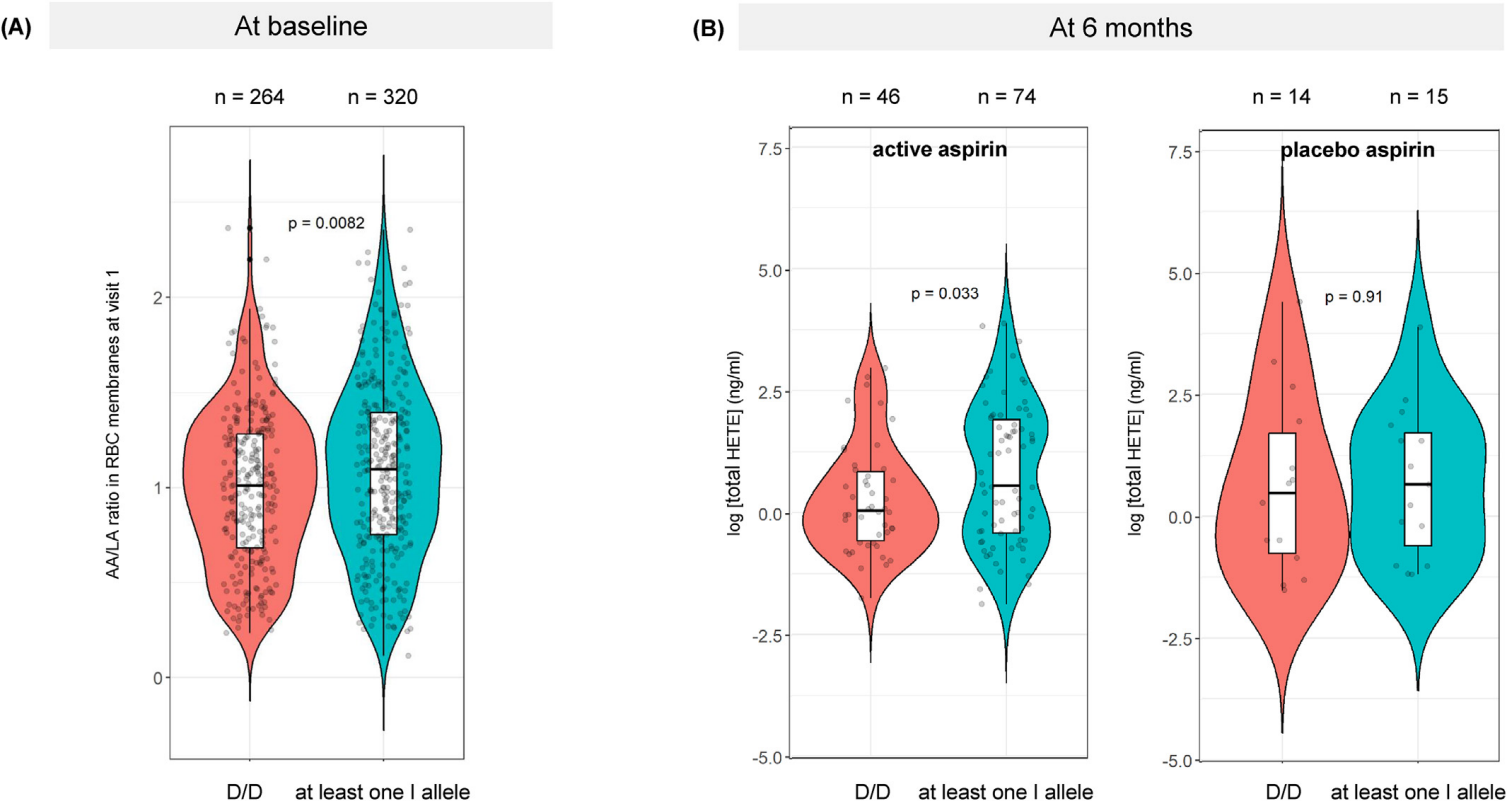
Comparison of the arachidonic acid/linoleic acid (AA/LA) ratio in red blood cell (RBC) membranes (A) and plasma total 15-hydroxyeicosatetraenoic acid (HETE) concentration (B) according to *fatty acid desaturase* insertion-deletion genotype. (A) Violin plot, with embedded box and whisker plot and individual data points, of baseline (seAFOod trial visit 1) RBC membrane AA/LA ratio values in 584 trial participants with a *fatty acid desaturase* insertion-deletion genotype and available RBC highly unsaturated fatty acid data [6]. The AA/LA ratio was calculated using the percentage levels of AA and LA of total fatty acids in red blood cell membranes measured by liquid chromatography-mass spectrometry [17,23]. *P* = 0.008 for the difference between I carriers and D/D homozygotes (Wilcoxon rank-sum test). (B) Violin plot, with embedded box and whisker plot and individual data points, of the plasma total (sum of *R*- and *S*- enantiomers) 15-HETE concentration after treatment with active aspirin or placebo aspirin for 6 mo [22]. *P* = 0.033 for the difference in plasma total 15-HETE concentration between I carriers and D/D homozygotes in the aspirin group only (t-test). AA/LA, arachidonic acid/linoleic acid; HETE, hydroxyeicosatetraenoic acid; RBC, red blood cell.

Consistent with our previous findings [11,12], individuals with ≥1 I allele exhibited a significantly higher RBC membrane AA/LA ratio than the participants with a D/D genotype (median: 1.10; IQR: 0.75–1.39 compared with 1.01 [0.68–1.28]; *P* = 0.008, Figure 2A). Trial participants with ≥1 I allele also had a higher plasma concentration of the aspirin-induced AA metabolite 15-HETE (mean: 0.79 ± 1.43 [SD]) than D/D individuals (0.28 ± 1.11; *P* = 0.03) after treatment for 6 mo, compatible with increased substrate bioavailability of AA in I allele carriers (Figure 2B). In keeping with elevated Δ5-desaturase activity on *n*–3 PUFAs, as well as *n–*6 PUFAs, the baseline EPA/ALA ratio in RBC membranes was also higher in participants with ≥1 I allele before the trial intervention (Supplemental Figure 1).

*FADS* Indel genotype and full colonoscopy outcome data were available for 528 seAFOod trial participants, which were distributed evenly across the trial treatment arms (Figure 1, Table 1). Clinical characteristics of the 528 seAFOod trial participants with *FADS* Indel genotype and colonoscopy outcome data were similar to the original *n* = 707 seAFOod trial population, for whom postrandomization trial data were available (Table 1) [6]. The distribution of *FADS* Indel genotypes was well balanced across the respective active and placebo intervention groups (percentage I carrier for active EPA [52.8%] compared with placebo EPA [57.7%]; active aspirin [56.6%] compared with placebo aspirin [54.0%]).

**TABLE 1:** Characteristics of 528 seAFOod trial participants with a *FADS* Indel genotype and complete colonoscopy data^1^

	Total study population	Placebos only	EPA only	Aspirin only	Aspirin and EPA	*P*^2^ (EPA vs. no EPA)	*P* (aspirin vs. no aspirin)	Excluded participants^3^	*P*^2^ (vs. excluded group)
No. of participants	528	138	123	136	131			179	
Age (y) (median [IQR^4^])	64.9 (6.3)	64.5 (7.8)	65.5 (6.2)	64.8 (6.3)	66.4 (7.7)	0.3	0.2	66.4 (6.2)	0.3
Sex						0.5	0.6		0.7
Male^5^	423 (80.1)	109 (79.0)	97 (78.9)	107 (78.7)	110 (84.0)			140 (78.2)	
Female	105 (19.9)	29 (21.0)	26 (21.1)	29 (21.3)	21 (16.0)			39 (21.8)	
BMI^6^						0.3	1.0		0.9
Underweight	2 (0.4)	0	1 (0.8)	1 (0.7)	0			1 (0.6)	
Normal	94 (17.9)	23 (16.8)	22 (18.2)	18 (13.2)	31 (23.7)			30 (16.8)	
Overweight	228 (43.4)	62 (45.3)	49 (40.5)	59 (43.4)	58 (44.3)			82 (45.7)	
Obese	201 (38.3)	52 (37.9)	49 (40.5)	58 (42.6)	42 (32.0)			66 (36.9)	
Diabetes						0.4	0.1		0.7
No	471 (89.2)	118 (85.5)	109 (88.6)	123 (90.4)	121 (92.4)			155 (86.6)	
Yes	57 (10.8)	20 (14.5)	14 (11.4)	13 (9.6)	10 (7.6)			24 (13.4)	
Tobacco smoking						0.4	0.4		0.5
Never	198 (37.5)	49 (35.5)	49 (39.8)	50 (36.8)	50 (38.2)			56 (31.3)	
Ever	252 (47.7)	64 (46.4)	66 (53.7)	65 (47.8)	57 (43.5)			95 (53.1)	
Current	78 (14.8)	25 (18.1)	8 (6.5)	21 (15.4)	24 (18.3)			14 (15.6)	
Alcohol intake^7^						0.8	0.5		0.8
None	80 (15.2)	23 (16.7)	19 (15.6)	19 (14.0)	19 (14.5)			30 (16.9)	
1–7 units/week	179 (34.0)	39 (28.3)	47 (38.5)	51 (37.5)	42 (32.1)			55 (31.2)	
8–21 units/week	161 (30.6)	45 (32.6)	29 (23.8)	44 (32.4)	43 (32.8)			53 (29.9)	
≥22 units/week	107 (20.2)	31 (22.5)	27 (22.1)	22 (16.2)	27 (20.6)			39 (22.0)	

1 Clinical characteristics of seAFOod trial participants were collected at entry to the trial [6].

2 The Wilcoxon test was used for the comparison of continuous variables, and the chi-squared test was used for the comparison of categorical variables

3 Excluded trial participants with either no *FADS* Indel genotype available or incomplete colonoscopy data.

4 IQR.

5 Values in brackets are the percentage value unless otherwise indicated.

6 BMI, body mass index. Underweight, <18.5 kg/m^2^; Normal, 18.5–24.9 kg/m^2^; Overweight, 25.0–29.9 kg/m^2^; Obese, ≥30 kg/m^2^). Missing BMI value for one case in the placebo group and 2 cases in the EPA only group.

7 Missing alcohol intake data for one case in the EPA only group. Missing alcohol intake data for 2 cases in the excluded participant group.

**FIGURE 3:**
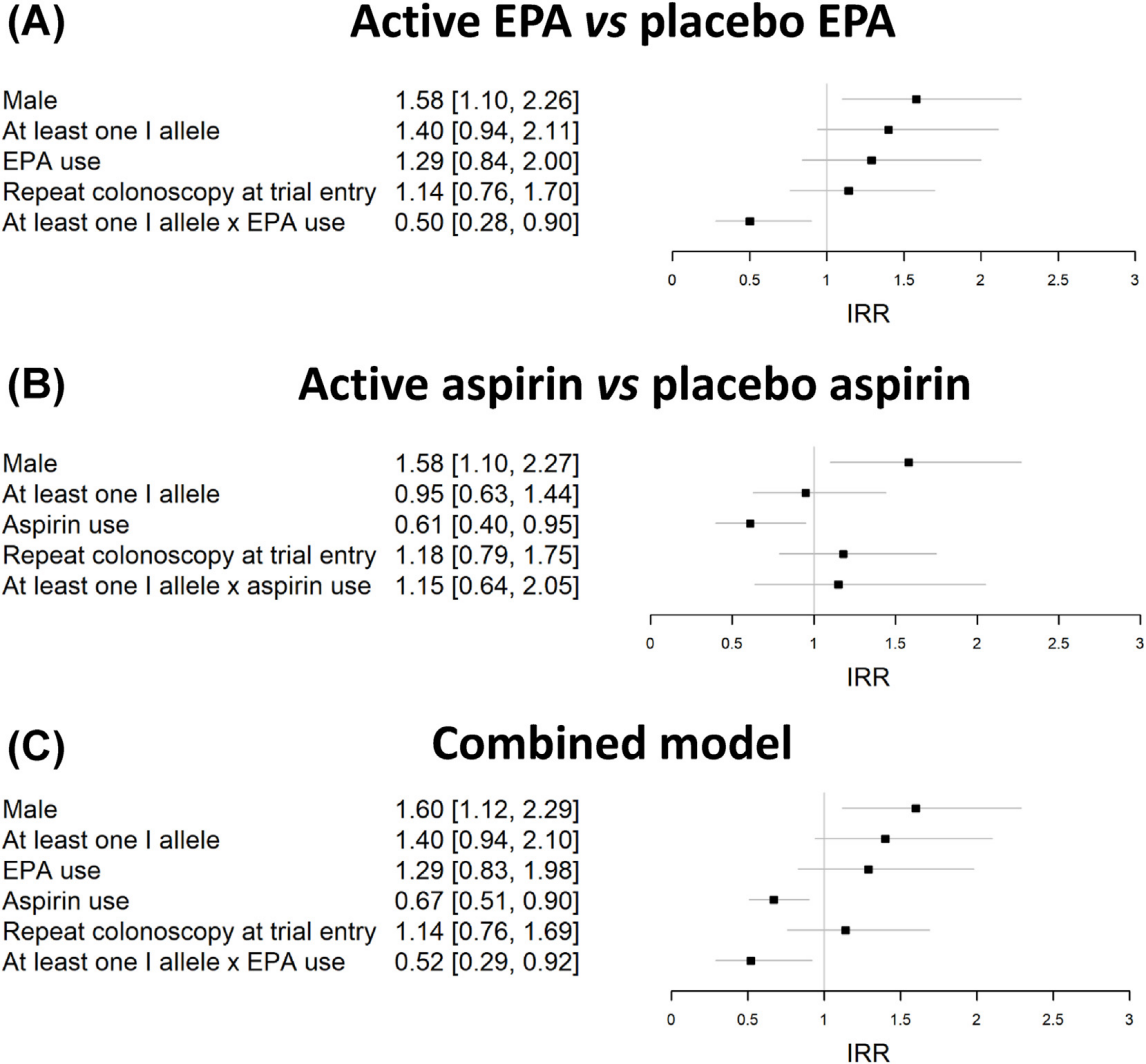
Multivariate models investigating the interaction between eicosapentaenoic acid (EPA) or aspirin treatment, and *fatty acid desaturase* insertion-deletion genotype (I carrier or not) for colorectal polyp number. Forest plots with the incidence rate ratio and 95% confidence interval (horizontal lines) for covariates for at the margins comparison by negative binomial regression of (A) active EPA vs. placebo EPA, (B) active aspirin vs. placebo aspirin, as well as (C) a combined model, which included both treatments as independent covariates. EPA, eicosapentaenoic acid.

EPA use, irrespective of *FADS* Indel genotype, was not associated with reduced total colorectal polyp number in the 528 individuals with a *FADS* Indel genotype (IRR: 0.92; 95% CI: 0.74, 1.16) inside a Poisson regression model, mirroring the primary analysis of the seAFOod trial [6]. In a negative binomial regression model that included *FADS* Indel genotype (I carrier or not), EPA use was also not associated with any significant change in colorectal polyp risk (IRR: 1.29; 95% CI: 0.84, 2.00) (Figure 3A). However, the presence of ≥1 I allele identified EPA users with a significant reduction in colorectal polyp number (IRR: 0.50; 95% CI: 0.28, 0.90) (Figure 3A). Consistent with higher AA levels and elevated tumorigenic risk, ≥1 *FADS* Indel I allele was associated with a trend toward increased colorectal polyp risk (IRR: 1.40; 95% CI: 0.94, 2.11) (Figure 3A).

By contrast, the same regression model according to aspirin use demonstrated the recognized polyp prevention efficacy of aspirin (IRR: 0.61; 95% CI: 0.40, 0.95) (Figure 3B), but there was no significant interaction between the *FADS* Indel genotype and aspirin use (IRR: 1.15, 95% CI: 0.64, 2.05) (Figure 3B), as expected if the primary mechanism of action of aspirin is COX inhibition, as opposed to limiting substrate (AA) availability (Figure 3B). The combined model with EPA and aspirin treatment as separate covariates did not materially change the key finding that EPA use in trial participants with ≥1 I allele displayed lower colorectal polyp risk (IRR: 0.52: 95% CI: 0.29, 0.92), comparable to the effect size associated with aspirin use (Figure 3C).

**FIGURE 4:**
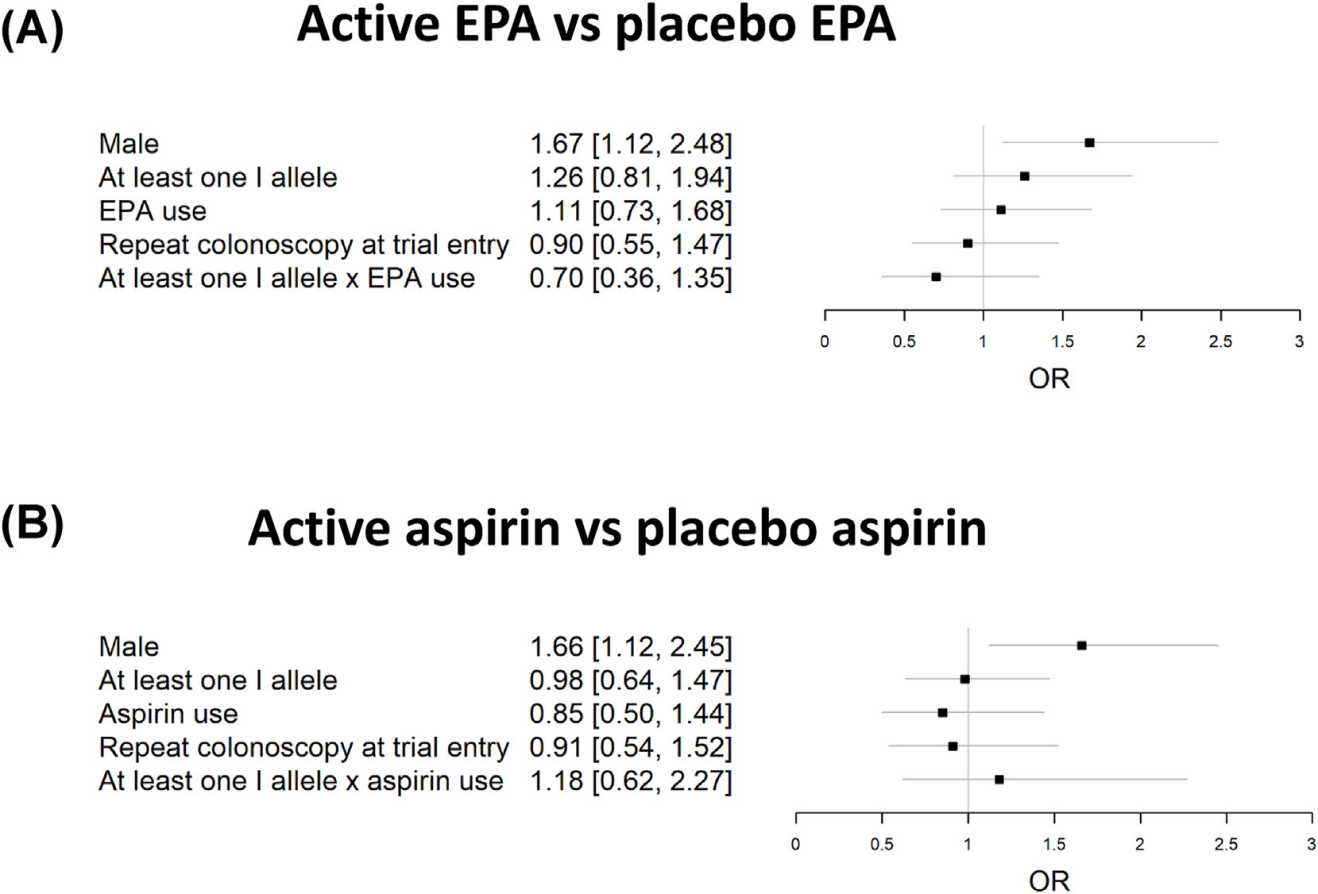
Multivariate models investigating the interaction between eicosapentaenoic acid (EPA) and aspirin treatment, and *fatty acid desaturase* insertion-deletion genotype (I carrier or not), for the colorectal polyp detection rate. Forest plots with the odds ratio and 95% confidence interval for covariates for at the margins comparison by logistic regression of (A) active EPA vs. placebo EPA, (B) active aspirin vs. placebo aspirin. EPA, eicosapentaenoic acid.

Logistic regression models of the PDR (the percentage number of individuals with one or more polyps) did not reveal a statistically significant association with any covariate, except male sex (Figure 4), although the point estimate for the OR for the I allele carrier × EPA interaction was consistent with the colorectal polyp number findings (compare with Figure 3).

## Discussion

We report that the *FADS* Indel, a functional 22 bp insertion-deletion polymorphism in *FADS2* (rs66698963), which is known to direct FADS1-dependent synthesis and availability of the *n*–6 HUFA AA, stratifies participants in the seAFOod trial that display colorectal polyp prevention by the *n–*3 HUFA EPA.

The elevated AA/LA ratio and higher levels of the AA-derived oxylipin metabolite 15-HETE in I allele carriers support the biological plausibility of 1) the association between the presence of ≥1 I allele and increased colorectal polyp risk in untreated individuals, driven by AA-mediated pro-tumorigenic signaling by PGE_2_ [7], and 2) the augmented polyp prevention efficacy of EPA treatment via antagonism of enhanced FADS1-dependent AA availability in I allele carriers [11]. We note that endogenous conversion of LA to AA can also be influenced at other steps along the HUFA synthesis pathway, including the elongation step mediated primarily by elongation of very long fatty acid 5 [27].

The gene (*FADS* Indel) × treatment interaction was specific for EPA, but not aspirin, consistent with current understanding of the mechanism of action of both agents, the latter being an irreversible inhibitor of COX-1 and COX-2 downstream of factors governing FADS1-dependent AA synthesis [28].

The molecular basis of the interaction between the *FADS* Indel genotype and EPA remains unclear. One possibility is that EPA competes more efficiently with AA for substrate availability leading to proinflammatory lipid mediator synthesis in individuals with one or more I alleles that have an increased propensity for FADS1-dependent AA synthesis [29]. An alternative hypothesis is that the *FADS* insertion polymorphism at rs66698963 modifies an interaction between EPA and the nearby SRE in intron 1 of *FADS2*, leading to decreased *FADS1* expression and function, as has been demonstrated for other agents such as simvastatin that modulate the function of SRE binding protein 1c [11]. Consistent with the possibility that alteration of SRE-mediated control of FADS1-dependent AA synthesis underlies the gene x treatment interaction that we observed, both EPA and the other key marine-derived bioactive *n–*3 HUFA DHA decrease expression of the SRE binding protein-1c in CaCo_2_ human CRC cells [30]. A key aim of future work will be to understand how the *FADS* Indel polymorphism alters cellular responses to EPA compared with DHA, an understanding of which will be critical for future precision use of mixed or single *n–*3 HUFA supplements.

Several single nucleotide polymorphisms (SNPs) in the *FADS* gene cluster (including rs174537, rs174546, and rs174547) have previously been reported to be associated with increased AA levels or an elevated Δ5-desaturase product (AA)-precursor (LA or DGLA) ratio [[31], [32], [33], [34]]. A small, randomized trial of 2.5 g mixed *n–*3 HUFAs for 6 mo has not revealed any interaction between the *FADS1* SNP rs174537 and rectal epithelial cell proliferation or apoptosis markers [35]. Further investigation of whether other genetic *FADS* variants that are associated with increased AA levels predict EPA efficacy is required, in addition to the understanding of the relation between SNPs in the *FADS* gene region and the rs66698963 genotype. Insertion-deletion polymorphisms are less well characterized than SNPs, in part, because high-throughput genotyping methods are not available. Our discovery of the *FADS* Indel emerged from a purposeful search for a genetic link to altered AA levels in human cells, which revealed a putative mechanism involving binding to the SRE in *FADS2* [11]. Subsequent studies in free-living humans showed that the major PUFAs affected by the *FADS* Indel genotype are DGLA and AA, and the downstream products of AA (adrenic acid [22:4*n–*6] and, to a limited extent, 22:5*n–*6 docosapentaenoic acid [DPA]), presumably via enhanced availability of AA substrate [36]. The same study (*n* = 1504 participants) had sufficient power to show similar trends for conversion of ALA to EPA, as well as downstream products 22:5*n–*3 DPA and DHA [36]. However, the overall ratio of AA to EPA+DHA increased from D/D to I/I individuals by 32% in this Chinese population, despite the apparent in vitro preference of FADS1 for *n–*3 over *n–*6 HUFAs. We conclude that the major effect of the *FADS* Indel is to increase AA on an absolute basis, as well as the percentage of total fatty acids and eicosanoid signaling precursors.

It is notable that the polyp prevention effect size of EPA in I allele carriers was similar to that of aspirin, for which there is already a robust body of evidence supporting its anti-CRC activity [37]. Therefore, *FADS* Indel genotype is a promising candidate biomarker for a precision medicine approach to CRC prevention by identification of individuals who would benefit most from supplemental and/or high dietary *n–*3 HUFA intake. The *FADS* Indel genotype may also predict disease risk by identifying the ‘high AA synthesizer’. Although *FADS1* expression drives AA synthesis, thereby influencing the replacement of AA in circulating and cellular pools as it is metabolized [11,38], variability in circulating AA levels within a genotype is also influenced by the balance and amount of dietary HUFAs [39,40]. Therefore, measurement of the AA level alone is unlikely to capture the impact of the *FADS* Indel genotype on disease risk.

This *post hoc* trial analysis of the seAFOod trial was limited by a lack of racial diversity in the study cohort that was predominantly White British and male [6]. The seAFOod trial cohort with full colonoscopy data only contained 54 (10%) I/I homozygotes, which is consistent with data on other groups of White-European descent [13], thereby restricting our ability to investigate the I allele copy number effect on the gene x treatment interaction for colorectal polyp risk reduction. By contrast, the *FADS* Indel I allele frequency is much higher in African and African-American populations (0.68) than in European groups (0.33) [41]. Moreover, it is recognized that African ancestry populations exhibit a high frequency of a *FADS* haplotype that is associated with efficient AA synthesis from dietary *n–*6 PUFAs [33]. These data predict that the *FADS* Indel I allele has been under positive selection pressure for high AA synthesis in ancestries with low animal/seafood intake. However, challenged with modern diets rich in LA, it is plausible that propensity for high AA synthesis predisposes to inflammation-mediated disease, if not balanced by *n–*3 HUFA availability.

Consistent with these observations, the VITAL randomized 2 x 2 factorial trial of mixed *n–*3 HUFAs (1 g daily) and vitamin D_3_ has reported differential benefit of *n–*3 HUFAs for reduction in myocardial infarction and colorectal polyp risk in African Americans compared with other ethnic groups [42,43]. These latter findings suggest that genetic factors governing FADS1-dependent AA synthesis may also influence cardiovascular disease risk reduction by dietary or supplemental *n–*3 HUFA intake, a hypothesis consistent with the emerging role of proinflammatory lipid mediator signaling in cardiovascular disease [44]. Our findings should prompt validation of the *FADS* Indel at rs66698963 as a treatment response biomarker in large intervention trials of cardiovascular disease risk reduction by *n–*3 HUFAs that have also collected participant DNA, such as VITAL and REDUCE-IT [42,45].

In conclusion, our data indicate that the *FADS* Indel at rs66698963 is a key functional polymorphism that predicts *n–*3 HUFA efficacy for colorectal polyp risk reduction in a randomized trial of EPA. Following validation of a role for *FADS* Indel genotype as a disease risk and/or therapeutic response biomarker in other diverse populations and health settings, stratification by genotype holds great promise for a precision approach to nutritional and therapeutic prevention of noncommunicable diseases by *n–*3 HUFAs [14].

## Acknowledgments

This work was part of the STOP-ADENOMA project (NIHR128210), which was funded by the Efficacy and Mechanism Evaluation Program, an MRC and NIHR partnership. The funder played no role in the research project including writing or the decision to submit the work for publication.

MAH is a NIHR Senior Investigator. MAH is supported by Cancer Research UK grant C23434/A24939.

We wish to thank all the participants and research staff who contributed to the seAFOod polyp prevention trial. STOP-ADENOMA collaborator Dr Amy Downing (University of Leeds) provided helpful advice.

## Author contributions

The authors’ responsibilities were as follows – KSDK, JTB, RCB, MAH: conceived and designed the study; MAH: gained approvals for the study, GS, YNL, JD, RCB, KSDK, JTB, MAH: were involved in the acquisition, analysis, or interpretation of data; GS, KSDK, JTB, MAH: drafted the manuscript; MAH: obtained funding for the study; all the authors: contributed to the critical review; and all authors: read and approved the final manuscript.

## Conflict of interest

The authors report no conflicts of interest.

## Data availability

De-identified seAFOod trial meta-data are available upon request to the Chief Investigator and the trial Sponsor (University of Leeds).
