# Supplementary material for: Fatty acid desaturase insertion-deletion polymorphism rs66698963 predicts colorectal polyp prevention by the n–3 fatty acid eicosapentaenoic acid: a secondary analysis of the seAFOod polyp prevention trial
Source: Am J Clin Nutr. 2024 Jun 13;120(2):360–8. doi: 10.1016/j.ajcnut.2024.06.004 (PMC11347814; doi:10.1016/j.ajcnut.2024.06.004)
Supplement: Multimedia component 1 [file mmc1.pptx]

## Slide 1
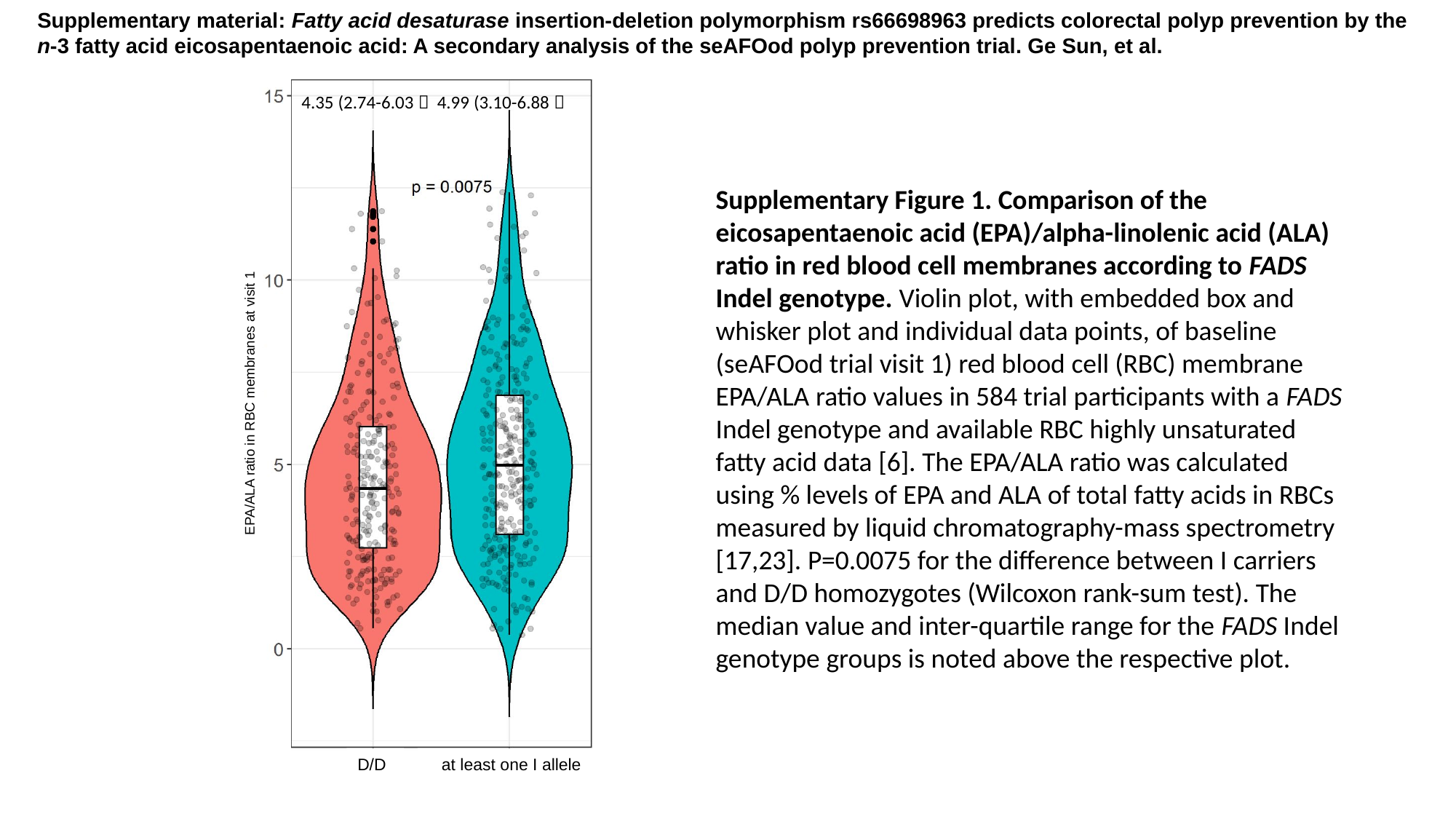

Supplementary material: Fatty acid desaturase insertion-deletion polymorphism rs66698963 predicts colorectal polyp prevention by the n-3 fatty acid eicosapentaenoic acid: A secondary analysis of the seAFOod polyp prevention trial. Ge Sun, et al.
4.35 (2.74-6.03）
4.99 (3.10-6.88）
EPA/ALA ratio in RBC membranes at visit 1
D/D
at least one I allele
Supplementary Figure 1. Comparison of the eicosapentaenoic acid (EPA)/alpha-linolenic acid (ALA) ratio in red blood cell membranes according to FADS Indel genotype. Violin plot, with embedded box and whisker plot and individual data points, of baseline (seAFOod trial visit 1) red blood cell (RBC) membrane EPA/ALA ratio values in 584 trial participants with a FADS Indel genotype and available RBC highly unsaturated fatty acid data [6]. The EPA/ALA ratio was calculated using % levels of EPA and ALA of total fatty acids in RBCs measured by liquid chromatography-mass spectrometry [17,23]. P=0.0075 for the difference between I carriers and D/D homozygotes (Wilcoxon rank-sum test). The median value and inter-quartile range for the FADS Indel genotype groups is noted above the respective plot.
